# Insights into the susceptibility of *Pseudomonas putida* to industrially relevant aromatic hydrocarbons that it can synthesize from sugars

**DOI:** 10.1186/s12934-023-02028-y

**Published:** 2023-02-02

**Authors:** Ana García-Franco, Patricia Godoy, Estrella Duque, Juan Luis Ramos

**Affiliations:** Estación Experimental del Zaidín. Consejo Superior de Investigaciones Científicas, c/Profesor Albareda nº 1, 18008 Granada, Spain

## Abstract

*Pseudomonas putida* DOT-T1E is a highly solvent tolerant strain for which many genetic tools have been developed. The strain represents a promising candidate host for the synthesis of aromatic compounds—opening a path towards a green alternative to petrol-derived chemicals. We have engineered this strain to produce phenylalanine, which can then be used as a raw material for the synthesis of styrene via *trans*-cinnamic acid. To understand the response of this strain to the bioproducts of interest, we have analyzed the in-depth physiological and genetic response of the strain to these compounds. We found that in response to the exposure to the toxic compounds that the strain can produce, the cell launches a multifactorial response to enhance membrane impermeabilization. This process occurs via the activation of a *cis* to *trans* isomerase that converts *cis* unsaturated fatty acids to their corresponding *trans* isomers. In addition, the bacterial cells initiate a stress response program that involves the synthesis of a number of chaperones and ROS removing enzymes, such as peroxidases and superoxide dismutases. The strain also responds by enhancing the metabolism of glucose through the specific induction of the glucose phosphorylative pathway, Entner-Doudoroff enzymes, Krebs cycle enzymes and Nuo. In step with these changes, the cells induce two efflux pumps to extrude the toxic chemicals. Through analyzing a wide collection of efflux pump mutants, we found that the most relevant pump is TtgGHI, which is controlled by the TtgV regulator.

**Graphical Abstract:**

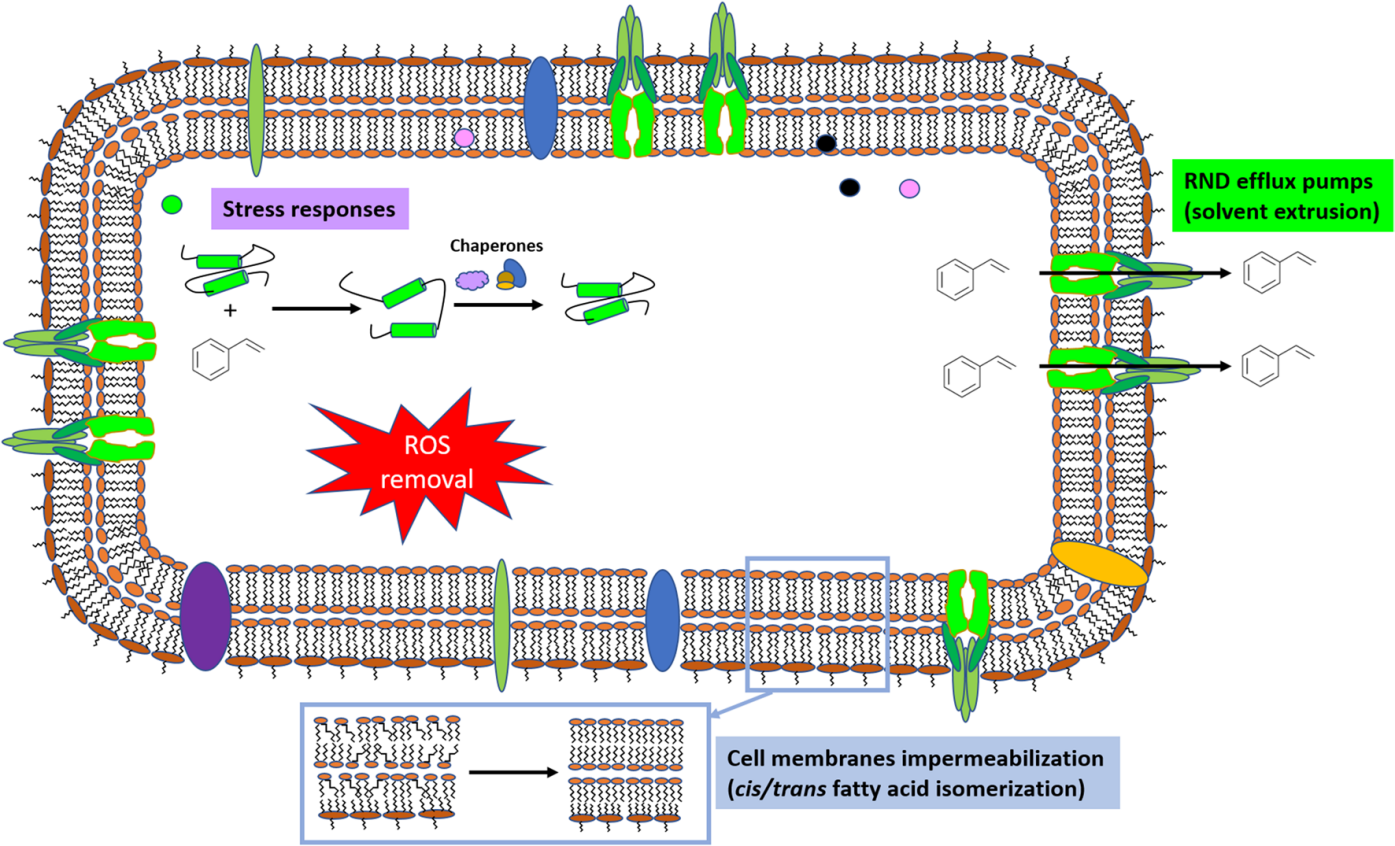

**Supplementary Information:**

The online version contains supplementary material available at 10.1186/s12934-023-02028-y.

## Introduction

Currently, an immense volume of petrol-derived chemicals are used for synthesis of polymers, detergents, textiles, fuels, pharma, and more [1–4]. With demand for these chemicals increasing, and limitations for fossil fuel resources mounting—combined with a global awareness of the climate crisis—there is a drive toward the sustainable, bio-based production of chemicals from renewable feedstocks [5–7]. Efforts to produce added-value chemicals through microbial fermentation are gaining momentum because the process offers a series of advantages over chemical synthesis; namely, the process (i) uses sustainable feedstocks, (ii) it can be operated at room temperature and ambient pressure, (iii) it can be easily scaled to an industrial level, and (iv) in most cases it can be used to produce highly pure compounds [8]. In order to fulfill cost-effectiveness prerequisites, the process requires a microbial cell platform that can efficiently and robustly generate a diverse range of chemicals with high yields in large-scale industrial processes [6, 9–12]. To achieve this requirement and to advance fermentation technology, researchers are using in silico metabolic flux predictions along with metabolic engineering as part of the so-called design-build-test-learn (DBTL) cycle [13]. We have used the DBTL cycle to develop a platform for the biosynthesis of aromatic compounds, including a range of industrial chemicals. Synthesis of aromatic compounds via chemical synthesis is highly energy intensive and requires toxic catalysts. Conversely, the synthesis of aromatic chemicals from bio sources requires the generation of aromatic amino acids (e.g., phenylalanine, tyrosine or tryptophane) as starting materials [1, 14–22].

In previous studies, it was shown that *Escherichia coli* and *Saccharomyces cerevisiae* bearing genes encoding a phenylalanine ammonia lyase and a *trans-*cinnamic decarboxylase enable them to produce styrene from phenylalanine via *trans-*cinnamic acid. However, production capability of this process is limited by the internal level of phenylalanine and the intrinsic styrene tolerance of the producer strains [23–26]. These studies revealed that, in addition to requiring genes to enable the metabolic steps, the chassis must be able to grow and thrive in the presence of the intermediates and final products being synthesized.

Pseudomonads are a promising chassis for the bioproduction of chemicals. They display unique characteristics needed for the production of aromatic compounds, including tolerance to toxic chemicals [16, 27–30]. *Pseudomonas putida* DOT-T1E was described by our group as a microorganism with extremely high tolerance to organic solvents [1, 28, 31, 32]. Genomic analysis of this strain revealed a versatile metabolic landscape that can be engineered to synthesize various chemicals [33].

We have previously shown that CM12-5, a derivative of DOT-T1E, is able to produce 0.5–1 g/L phenylalanine when glucose is supplied as a single carbon source or as part of a mixture of chemicals that originate from the 2G hydrolysis of lignocellulosic biomass [11, 14]. The present study was conceived to provide insights into the tolerance of *Pseudomonas putida* DOT-T1E to *trans-*cinnamic acid and styrene. To achieve this, we used omics approaches to elucidate the response of wild-type and mutant strains to elucidate the response of this strain to these chemicals in batch culture or after the sudden addition of the test compounds. By identifying the key molecular determinants of tolerance to these chemicals, this work sheds light on the response of this microorganism to aromatic chemicals of industrial interest.

## Experimental procedures

### Bacterial strains and growth conditions

*Pseudomonas putida* DOT-T1E was grown on M9 minimal medium with glucose 5 g l^−1^ [34] as the sole carbon source. When required, 10 mM *trans-*cinnamic acid and/or styrene (through the gas phase) were supplied. Cultures were incubated at 30 °C and shaken on an orbital platform at 200 strokes per minute.

### Growth parameters

To determine growth rate, overnight cultures of *P. putida* were harvested by centrifugation (13000 g, 5 min) and washed once with M9 minimal medium [34]. Cells were suspended to an OD_660_ of ~ 0.1 in 25 ml of the test medium in 250 ml conical flasks. Doubling times were determined during exponential growth as a slope of the data points obtained by plotting CFU ml^−1^ against time.

To determine cell dry weight, samples of cultures were transferred into 2 ml pre-weighted Eppendorf tubes and pelleted at 13000 g for 10 min. The pellets were washed once with M9 medium and left to dry at 70 °C for 48 h. Substrate consumption rates and specific carbon consumption rates were determined during the initial 24 h of culture as described by Dvorák and de Lorenzo [35]. Cell growth was routinely monitored at 660 nm using a UV-1900i UV–vis spectrophotometer (Shimadzu, USA).

### *Pseudomonas putida *DOT-T1E tolerance to *trans-*cinnamic acid and styrene over the long term

Overnight cultures were diluted to an OD_660_ of 0.1 and supplemented with different concentrations of *trans-*cinnamic acid (0, 15, 25, 50, 75 and 90 mM) or styrene (0.1 and 1% (v/v)) and grown and monitored under these conditions for 24 h. The number of viable cells was determined before aromatic compounds were added and after 4, 6 and 24 h.

### Survival in response to *trans-*cinnamic acid or styrene sudden shock

Overnight cultures were diluted to an OD_660_ of 0.1 and grown under the same conditions until the cultures reached a turbidity of about 0.8 at 660 nm. Then the cultures were divided into three aliquots: to the first aliquot, 0.1% (v/v) styrene was added; to the second aliquot, 50 mM *trans-*cinnamic acid was added; and the last aliquot was kept as a control. The number of viable cells was determined before aromatic compounds were added and 10, 30 and 60 min later.

### Analysis of phospholipids

Phospholipids were extracted by the method described by Bligh and Dyer [36], then saponified and esterified as described by Junker and Ramos [37]. The fatty acids were identified by mass spectrometry after gas chromatographic separation.

### Transcriptomics

To study the *P. putida* DOT-T1E transcriptome under different conditions, the strain was grown overnight in M9 minimal medium with glucose 5 g l^−1^ in the absence and in the presence of 10 mM *trans-*cinnamic acid and styrene supplied through the gas phase. On the following day, cultures were diluted to an OD_660_ of 0.1 and grown under the same conditions until the cultures reached the end of the exponential phase. Cells (20 ml) were harvested by centrifugation (5000 g for 10 min at 4 ºC) and stored at − 80 ºC until processing. Three independent biological replicates were prepared.

RNA isolation was carried out according to TRIzol Reagent instructions (Invitrogen). Extracts were treated with RNase-free DNase. The integrity of total RNA and the presence of DNA contamination were assessed with a NanoDrop One. DNA removal was carried out via treatment with Nucleo-Spin RNA Plant (Macherey–Nagel). The integrity and quality of total RNA was assessed with an Agilent 2100 Bioanalyzer (Agilent Technologies). Removal of rRNA was performed using RiboZero rRNA removal (bacteria) kit from Illumina, and libraries of 100-bp single-end reads were prepared using a TruSeq Stranded Total RNA Kit (Illumina). Libraries were sequenced using a NextSeq550 sequencer (Illumina).

The raw reads were pre-processed with SeqTrimNext [38] using the specific NGS technology configuration parameters. This pre-processing keeps the longest and removes sequences below 25 bp. It also removes low-quality, ambiguous and low-complexity stretches, linkers, adapters, vector fragments, and contaminated sequences. Clean reads were aligned and annotated using the Pcl and Bamy reference genomes with Bowtie2 [39] in BAM files, which were then sorted and indexed using SAMtools v1.484 [40]. Uniquely localized reads were used to calculate the read number value for each gene via Sam2counts (https://github.com/vsbuffalo/sam2counts). DEgenes Hunter was used to analyze differentially expressed genes (DEGs), which provides a combined p value calculated (based on Fisher’s method) using the nominal p values provided by from edgeR [41] and DEseq2 [42]. This combined p value was adjusted using the Benjamini-Hochberg (BH) procedure (false discovery rate approach) and used to rank all the obtained DEGs. For each gene, significance thresholds were established by combining p value < 0.05 and log2-fold change > 2 or <  − 2. The annotated DEGs were used to identify the Gene Ontology functional categories and KEGG pathways. Gephi software (https://gephi.org) was used to generate the DEG networks [43].

### Proteomics

Proteomic analysis was carried out at the proteomic facility of CNB-CSIC (Madrid. Spain; http://proteo.cnb.csic.es/proteomical) using the iTRAQ procedure [44, 45]. To study the proteome of *P. putida* DOT-T1E, cells were cultured as above except that 30 ml of cultures with a turbidity of about 1 at 660 nm were harvested by centrifugation (5000 g for 10 min at 4 ºC), then washed twice with M9 medium. Cell pellets were then stored at − 80 °C. For the preparation of protein extracts, cell pellets were suspended in 600 µL of lysis buffer [45], and lysis was carried out at 4 °C by sonication applying a 40 J dose with amplitude of vibration of 30% and pulses of 10 s followed by resting intervals of 5 s. To remove cellular debris lysates were centrifuged for 20 min at 14 000 × *g* at 4 °C to remove cellular debris. Protein (15 μg) was treated with 100 mM Tris (2-Carboxyethyl) phosphine hydrochloride (TCEP) to reduce disulfide bridges, alkylated with 200 mM chloroacetamide (CAA) and digested with trypsin. Upon peptide separation samples were labelled with the TMT 8-plex or TMT 11-plex reagent (one label per sample). After 2 h labelling samples were cleaned using a reverse C18 BoncElute Agilent column.

Protein identification and the analysis of differential expression were performed by Proteobiotics (Madrid, Spain). Peak lists were generated with the Mascot Daemon (version 2.5.1), OMSSA 2.1.9, X!TANDOM 23.02.01.1 and Myrimatch 2.2.140 softwares. The mgf files from each sample were merged and MS/MS spectra assigned using the non‐redundant RefSeq protein entries for *P. putida* comprising 5313 protein sequences totaling 1,656,176 amino acids (National Center for Biotechnology Information download, 2021). We identified 1960 proteins and the quantification revealed differential expression of 63 proteins in *trans*-cinnamic acid versus 163 proteins in styrene. In the presence of the two aromatic compounds 305 proteins had differential level. The search was performed using the following criteria: tryptic peptides with a maximum of two miss cleavages, mass tolerances of 25 ppm on the parent ion and 0.02 Da on the MS/MS, fixed modification for carbamidomethylated cysteine and variable modification for methionine oxidation. Peptides were identified with a *q*‐value threshold below 0.05. Proteins were considered validated when at least two distinct peptides were detected. The false discovery rate for protein identification was estimated with a reversed decoy database to be less than 1%. Proteins showing an abundance change equal or higher than 1.5 (log_2_ fold change) and a *q*-value equal or lower than 0.05 were eventually considered as differentially abundant [45].

### DNA techniques

DNA was manipulated using standard laboratory protocols [46, 47]. Genomic DNA was isolated using the Wizard Genomic DNA purification Kit (Promega USA), while plasmid DNA was isolated with the QIAprep Spin Miniprep kit (Qiagen, USA). DNA concentration was measured with a NanoDrop One (Thermo Scientific, USA). PCR DNA amplification was performed with appropriate primers (Additional file 4: Table S1), dNTPs and Phusion High-Fidelity DNA polymerase (Thermo Scientific, USA) or Taq DNA polymerase (Roche, Germany), as recommended by the manufacturers.

### Electroporation

Electroporation of *Pseudomonas putida* DOT-T1E was performed as described elsewhere [46, 47], using a MicroPulser electroporator and Gene Pulser Cuvettes with 0.2 cm gap (Bio-Rad, USA). Transformants were selected on LB agar plates with kanamycin (25 μg ml^−1^) and incubated at 30 °C for 24 to 36 h.

### Construction of stress response-deficient mutants

Inactivation of *sucC, pflU and arcA* was achieved as described by Godoy et al. [14]: a DNA fragment spanning the central part of these ORFs was amplified by PCR from *P. putida* DOT-T1E genomic DNA using appropriate primers (Additional file 4: Table S1). The resulting amplified DNA was cloned into pMBL-T [48] to yield pMBL-T::sucC, pMBL-T::pflU and pMBL-T::arcA, respectively. These plasmids were digested with BamHI and then ligated to the BamHI kanamycin Ω-interposon fragment from plasmid pHP45ΩKm. The resulting chimeric plasmids were named pMBL-T::sucCΩKm, pMBL-T::pfluΩKm and pMBL-T::arcAΩKm, respectively. These plasmids were individually electroporated into *P. putida* DOT-T1E and putative Km^R^ recombinant mutants were selected on kanamycin LB plates. A number of Km^R^ clones were retained, and Southern blotting was used to verify the insertional mutation in the respective gene (data not shown).

### Glucose concentration in culture supernatants

For determination of glucose, cultures (1 ml) were centrifuged (13000 g for 10 min at 4 °C), and supernatants were stored at − 20 °C until analyzed. For analysis of glucose, the D-glucose-HK Assay Kit was used according to the manufacturer’s instructions. Measurements were performed using a TECAN Sunrise 200 microplate absorbance reader.

## Results

### Growth of DOT-T1E in the presence of different concentrations of *trans-*cinnamic acid in liquid medium containing or not styrene in the gas phase

The genomic analysis of DOT-T1E indicated that this strain lacks the PAL enzyme for the conversion of phenylalanine into *trans-*cinnamic acid and the corresponding *trans-*cinnamic acid decarboxylase required to produce styrene [33]. A number of *Pseudomonas* strains have been described that can use styrene as the sole C-source via the styrene monooxygenase pathway. In this pathway, the aromatic hydrocarbon is oxidized to produce styrene oxide, which is subsequently metabolized to phenylacetaldehyde and phenylacetic acid before it is directed to central metabolism [49]. The metabolic analysis of the genome of this strain revealed that DOT-T1E lacks the styrene oxidation system [33]. We found, as expected, that DOT-T1E does not grow with styrene when this compound was supplied as the sole C-source.

Although strains of *P. stutzeri* have been described that can use cinnamic acid as the sole C-source [50], no such findings have been published for *P. putida*. To confirm that *P. putida* DOT-T1E does not use *trans-*cinnamic acid as a C source to produce biomass, we inoculated *P. putida* DOT-T1E in M9 minimal medium with 10 mM *trans*-cinnamic acid as the sole C-source at an initial turbidity of 0.05 at 660 nm, and monitored culture turbidity for 72 h. No growth was observed. To determine if these aromatic compounds affect growth, we set up cultures with glucose and 10 mM *trans-*cinnamic acid or styrene supplied through the gas phase and determined growth rates, cell density and yields of the cultures (Table 1). We found that in the glucose medium the strain grew exponentially until reaching high cell density (Table 1) and a biomass yield of 0.4 ± 0.01 g/g glucose. Doubling time in this medium with glucose was around 90 min (Table 1). Growth with glucose as the carbon source was not halted in the presence of 10 mM *trans-*cinnamic acid or styrene in the gas phase although the growth rates and yields were slightly lower in the presence of the aromatics (Table 1), suggesting that the strain needed to expend energy in order to thrive in the presence of the chemicals. The growth yield in the presence of either aromatic compound was lower that without (i.e., 0.39 to 0.32 g/g glucose). This, therefore, confirms that DOT-T1E is a potential host for the biotransformation of phenylalanine into styrene via *trans-*cinnamic acid; however, production conditions will need to be optimized to decrease the intrinsic negative effects associated with the noxious nature of the chemicals.

**Table 1:** Growth characteristics of *P. putida* DOT-T1E with glucose in the absence and in the presence of *trans*-cinnamic acid and styrene in the gas phase

Compounds in the culture medium	tg	CFU ml^−1^_max_	Y
Glucose	90.8 ± 0.0	6.67E + 09 ± 1.18 + 08	0.40 ± 0.01
Glucose + styrene (g)	88.5 ± 20.7	2.28E + 09 ± 1.53E + 08	0.39 ± 0.02
Glucose + 10 mM tCA	96.5 ± 5.9	5.33E + 09 ± 1.30E + 09	0.33 ± 0.01
Glucose + 10 mM tCA + styrene (g)	96.8 ± 3.2	1.48E + 09 ± 2.71E + 08	0.32 ± 0.01

*tg* generation time (min), CFU ml^−1^_max_ maximum colony forming units, *Y* yield (g cells g^−1^ sugar consumed in 24 h). The data are the average of at least two independent assays done each in triplicate ± standard deviations

**Fig. 1:**
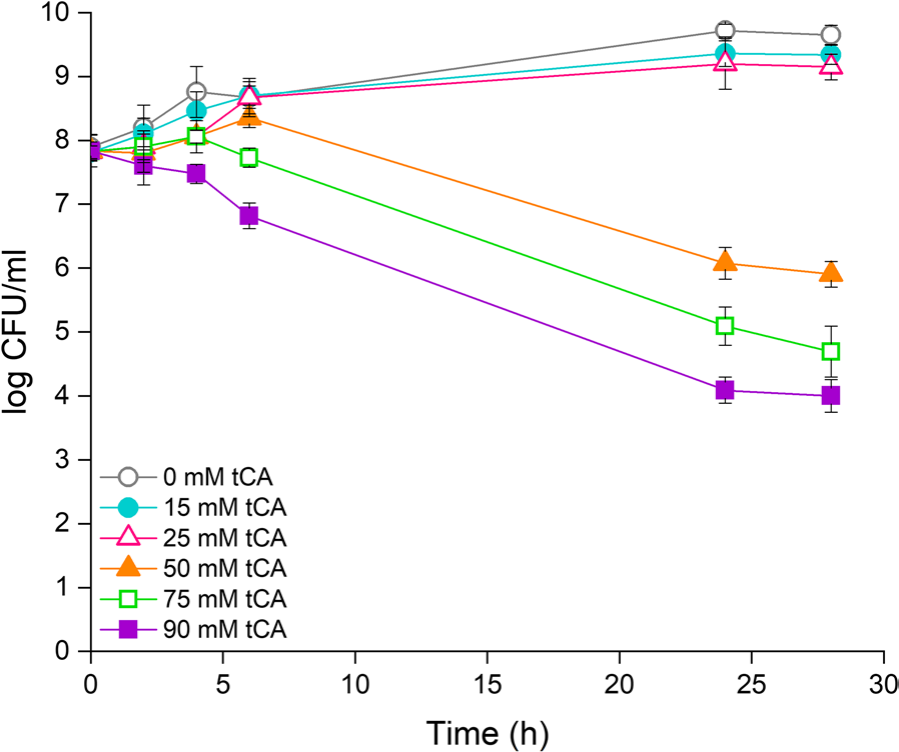
Tolerance of *P. putida* DOT-T1E to *trans-*cinnamic acid (0, 15, 25, 50, 75 and 90 mM) in M9 glucose. The results shown represent the averages and standard deviations of three independent assays. Control, grey open circles; or with addition of *trans*-cinnamic acid at 15 mM (blue solid circle); 25 mM (pink open triangles); 50 mM, (orange solid triangles); 75 mM, (green open squares); and 90 mM, (purple solid squares)

We tested growth of DOT-T1E in the presence of increasing concentrations of *trans-*cinnamic acid to determine its inhibitory potential. To this end we determined CFU/ml rather than conducting turbidity measurements, which would have given misleading results due to the ability of dead cells to affect turbidity. We found that concentrations below 25 mM *trans-*cinnamic acid did not result in halted growth (Fig. 1); however, concentrations above 50 mM not only prevented growth but also led to loss of cell viability after several hours of incubation. The number of CFU/ml decreased faster as the concentration of the acid increased in the medium (Fig. 1). It has been reported before that exposure of cells to subinhibitory concentrations of chemicals can induce defense mechanisms that lead to enhanced tolerance to the noxious chemicals [51, 52]. For this reason, *P. putida* DOT-T1E cells were pre-grown on glucose plus 10 mM *trans-*cinnamic acid to see if pre-adaptation to this aromatic acid occurs and results in enhanced tolerance. To this end cultures were transferred to the same medium but with 0, 15, 25, 50, 75 and 90 mM of the aromatic acid. Growth of the cells that were pre-exposed to *trans-*cinnamic acid was similar to the growth of cells that were not pre-exposed to the acid (Additional file 1: Fig. S1A).

We also tested whether cells pre-exposed to styrene in the gas phase could enhance tolerance to *trans-*cinnamic acid. As above, styrene pre-exposed cells behaved identically to cells that were not pre-exposed (Additional file 1: Fig. S1B). Similar results to those described above were obtained with *P. putida* DOT-T1E pre-grown with styrene and 10 mM *trans-*cinnamic acid in the liquid medium (Additional file 1: Fig. S1C).

Because styrene solubility in water is very low (310 mg/L), and *P. putida* DOT-T1E grows at saturating concentrations of styrene in water, the above series of assays cannot be performed for styrene. Instead, we analyzed the response of DOT-T1E growing in different culture media to sudden shocks of 0.1% (v/v) styrene or 50 mM of *trans-*cinnamic acid, as described below.

### Survival of DOT-T1E in response to *trans*-cinnamic or styrene shock

**Fig. 2:**
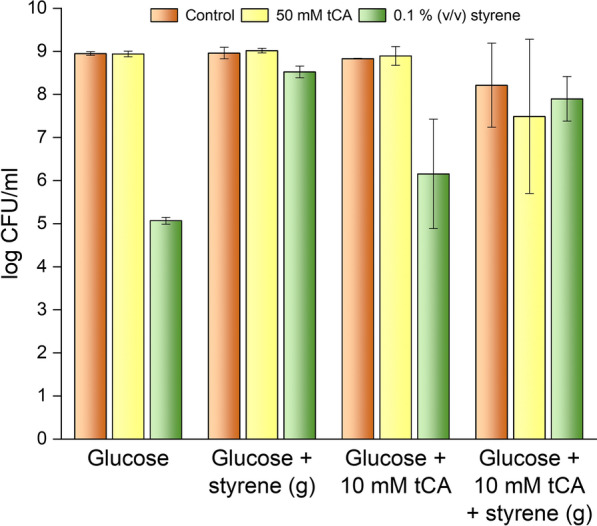
Survival of *P. putida* DOT-T1E to sudden shocks of *trans-*cinnamic acid (50 mM) and styrene (0.1% (v/v)) in cells grown on glucose, glucose plus styrene in the gas phase, glucose plus 10 mM *trans-*cinnamic or with the two test compounds simultaneously. The results shown are the averages and standard deviations of three independent assays of cells that survived a 60 min shock upon addition of 50 mM *trans*-cinnamic acid (yellow bars) or 0.1% (v/v) styrene (green bars). The control assay is represented in orange

In the following series of assays, we determined the survival in the short term of DOT-T1E grown on glucose, glucose plus 10 mM *trans-*cinnamic, glucose plus styrene in the gas phase, and with the two test compounds simultaneously. In this series of assays, cells were grown until the mid of the exponential phase; at that point, we made a sudden addition of 50 mM *trans-*cinnamic acid or 0.1% (v/v) styrene. We found that regardless of the growth conditions (i.e., glucose alone; glucose plus 10 mM *trans-*cinnamic; glucose with styrene in gas phase; and glucose with the two aromatic compounds), DOT-T1E exposed to 50 mM *trans*-cinnamic acid survived in the short term (i.e., 60 min) without a significant decrease in the number of viable cells (Fig. 2).

In another series of assays 0.1% (v/v) styrene was added to the liquid medium. For glucose-grown cells, upon styrene addition, only around 1 in 10^4^ CFU/ml survived the solvent shock (Fig. 2). This low number of viable cells remained constant for 60–180 min but began to recover after 4 h incubation, so that growth was restored (not shown). In contrast, when cells had been pre-adapted to styrene in the gas phase (in the absence or in the presence of *trans-*cinnamic acid) around 90% of cells survived. We also tested if pre-adaptation to 10 mM *trans-*cinnamic affected cell tolerance to a sudden styrene shock. We found an intermediate situation with 1 out 10^3^ cells surviving a sudden styrene shock (Fig. 2). These results suggest that this strain possesses a series of adaptive mechanisms for styrene, which are induced by either the aromatic hydrocarbon or the acidic aromatic compound.

Segura et al. [53] previously found that different *P. putida* strains exhibited differential sensitivity to toluene and xylenes. Of the tested strains *P. putida* KT2440 was particularly sensitive to toluene regardless of the growth conditions. We have now grown KT2440 in M9 minimal medium with glucose in the absence and in the presence of styrene supplied through the gas phase and tested survival upon a sudden shock of 0.1% styrene. We found that the survival rate of KT2440 grown on glucose was below 1 in 10^8^ CFU/ml (Fig. 2SA) and that the pre-growth in the presence of styrene had no effect on survival (Fig. 2SB). When the same assay was performed with *E. coli* ET8000, a potential chassis to produce styrene, survival to the sudden solvent shock was below 1 in 10^9^ CFU/mL (Additional file 2: Fig. S2). This makes DOT-T1E a *sine qua non* chassis for production of this toxic chemical.

In previous studies, we reported that cells grown with toluene in the gas phase survived a sudden shock of 1% (v/v) toluene [51]. We grew DOT-T1E cells on M9 minimal medium with glucose and toluene supplied in gas phase, and exposed these cells to sudden styrene shocks as above. We found that growth of DOT-T1E in the presence of toluene enabled the strain to survive styrene shocks and, as expected, what suggests that a series of common mechanisms are induced to protect cells against the presence of aromatic hydrocarbons. (not shown).

In *Pseudomonas* it is well stablished that in response to a number of insults (toxic organic chemicals, metals, antibiotics, etc.) cells fortify their membranes by activation of a constitutively made *cis–trans* isomerase that catalyzes the isomerization of *cis* unsaturated fatty acids to their corresponding *trans*-isomers [17, 37, 52, 54–57]. To confirm that styrene and *trans*-cinnamic acid provoked the general response to the chemicals under study, we determined *cis/trans* fatty acid ratios. The *cis/trans* ratio in glucose grown cells was 4.03 with C16:1,9 *cis* being the most abundant unsaturated fatty acid (about 23% of total) (Table 2). When cells were grown in the presence of *trans-*cinnamic acid, styrene or both compounds, the *cis/trans* ratio was lower and in the range of 2.9 and 1.5. Under these conditions, the level of C16:1,9 *cis* was almost as high as the corresponding *trans* isomers, and the ratio of *trans* C18,1:11 was two-fold higher than in the absence of stressors. These results support the notion that cells fortify cells membranes via a fast *cis to trans* isomerization in response to toxic aromatic compounds.

**Table 2:** Fatty acid composition of *P. putida* DOT-T1E cells grown on glucose in the absence or in the presence of *trans-*cinnamic acid (tCA), styrene supplied in the gas phase (g) or both aromatic compounds simultaneously (tCA + styrene (g)).

Fatty acid	Growth medium			
	Glucose	Glucose + 10 mM tCA	Glucose + styrene (g)	Glucose + 10 mM tCA + styrene (g)
C14:0	0.19 ± 0.01	0.20 ± 0.00	0.14 ± 0.02	0.15 ± 0.02
C15:0	0.04 ± 0.00	0.04 ± 0.01	0.11 ± 0.03	0.07 ± 0.00
C16:1,9 cis	23.14 ± 0.84	15.73 ± 1.10	19.51 ± 0.29	13.52 ± 2.89
C16:1,9 trans	10.49 ± 1.11	18.04 ± 0.97	12.56 ± 0.11	19.85 ± 0.88
C16:0	23.63 ± 0.44	23.77 ± 0.02	22.80 ± 1.11	23.45 ± 2.34
C17:cyclo	4.96 ± 0.14	5.68 ± 0.00	4.61 ± 0.66	4.63 ± 0.43
C17:0	0.06 ± 0.00	0.06 ± 0.00	0.42 ± 0.11	0.17 ± 0.05
C18:1,11 cis	32.40 ± 0.17	28.55 ± 0.82	32.68 ± 0.14	28.05 ± 0.08
C18:1,11 trans	3.28 ± 0.48	6.23 ± 0.95	4.98 ± 0.28	7.65 ± 3.54
C18:0	1.75 ± 0.00	1.64 ± 0.01	2.11 ± 0.27	1.82 ± 0.26
C19:cyclo	0.00 ± 0.00	0.00 ± 0.00	0.00 ± 0.00	0.00 ± 0.00
TOTAL (%)	100	100	100	100
Cis/trans	4.03 ± 0.54	1.82 ± 0.22	2.97 ± 0.09	1.50 ± 0.34
Sat/insat	0.37 ± 0.01	0.37 ± 0.00	0.37 ± 0.01	0.37 ± 0.04

Values are the average and standard derivations of three assays

### Transcriptional and proteomic responses to *trans-*cinnamic acid and styrene

As a next step, we used omics approaches to analyze the specific responses of the strain to the stressors. Transcriptomic analyses were carried out in triplicate (see Volcano plots in Additional file 3: Fig S3) and the values were considered statistically different if there was a change of log2 ≥ 2.0 or log2 ≤ − 2.0 and a *p*-value < 0.05. Data were analyzed from different angles to extract as much information as possible. Tables 3 and 4 show that a limited number of genes were significantly up- or downregulated in response to the stressor. We found about 240 genes were overexpressed in response to styrene, 209 in response to *trans-*cinnamic acid, and 337 in response to the simultaneous presence of both aromatic compounds (Table 3). Regarding downregulated genes, we found that 129, 132 and 141 genes were expressed at lower levels in the presence of styrene, *trans-*cinnamic or both aromatic compounds, respectively (Table 4). It should be noted that a number of genes with unknown functions were also induced or repressed in response to either styrene or *trans*-cinnamic acid (Additional file 5: Tables S2, Additional file 6: Table S3 and Additional file 7: Table S4). Proteomic and transcriptomic analyses revealed similar trends although, in general, the fold change of the induced and repressed proteinswere lower. Below we analyze the transcriptomic data in detail and provide a summary of insights from the proteomic analysis.

**Table 3:** Number of genes upregulated of *P. putida* DOT-T1E in response to *trans-*cinnamic acid (tCA), styrene supplied in the gas phase or both aromatic compounds

Gene category	tCA	Styrene (g)	Styrene (g) + tCA
Amino acid and protein biosynthesis	40	24	59
Amino acid, peptide and protein metabolic process	0	6	6
Antiholins	2	2	1
Aromatic compound catabolic process	7	9	11
ATP synthesis	10	6	8
Cell division	0	6	0
Cell shape	4	1	2
DNA replication, recombination and repair	6	1	14
Efflux pump	10	10	11
Glyoxylate metabolic process	0	2	0
Krebs cycle	16	16	20
Lipid metabolic process	0	7	10
Membrane component	33	48	61
Oxidoreductase activity	4	1	0
Phosphate metabolism	0	1	1
Propionate metabolism	0	5	4
Respiration	24	24	23
Stress	17	21	41
Sugar uptake/metabolism	19	17	21
Sulfur metabolism	1	3	7
Transcription and translation	8	26	32
Urea cycle/nitrogen metabolism	8	4	5
TOTAL	209	240	337

**Table 4:** Number of genes downregulated of *P. putida* DOT-T1E in response to *trans-*cinnamic acid (tCA), styrene supplied in the gas phase or both aromatic compounds

Gene category	tCA	Styrene (g)	Styrene (g) + tCA
Amino acid and protein biosynthesis	5	2	3
Amino acid, peptide and protein metabolic process	24	19	17
Cell adhesion	0	1	1
Cell motility	3	3	2
Cell shape	5	4	4
Cell wall hydrolase	1	0	0
Efflux pump	2	3	3
Gluconeogenesis	1	1	1
Glyoxylate metabolic process	0	2	2
Krebs cycle	1	0	0
Lipid metabolic process	0	3	3
Membrane component	32	45	48
Nitrogen metabolism	1	0	1
Organic catabolic process	7	4	8
Polysaccharide biosynthetic process	0	1	2
Pyrroloquinoline quinone biosynthetic process	6	5	6
Respiration	2	2	5
Stress	9	8	5
Sugar uptake/metabolism	3	5	8
Transcription and translation	30	21	22
TOTAL	132	129	141

Although the number of genes within a specific set of functions varied among the control (glucose) and the three tested conditions, a similar gene upregulation trend in the presence of aromatic chemicals was observed for transmembrane proteins, and those involved in sugar uptake/metabolism, Krebs cycle, respiratory chains and ATP synthesis (Additional file 6: Tables S3 and Additional file 7: S4).

**Fig. 3:**
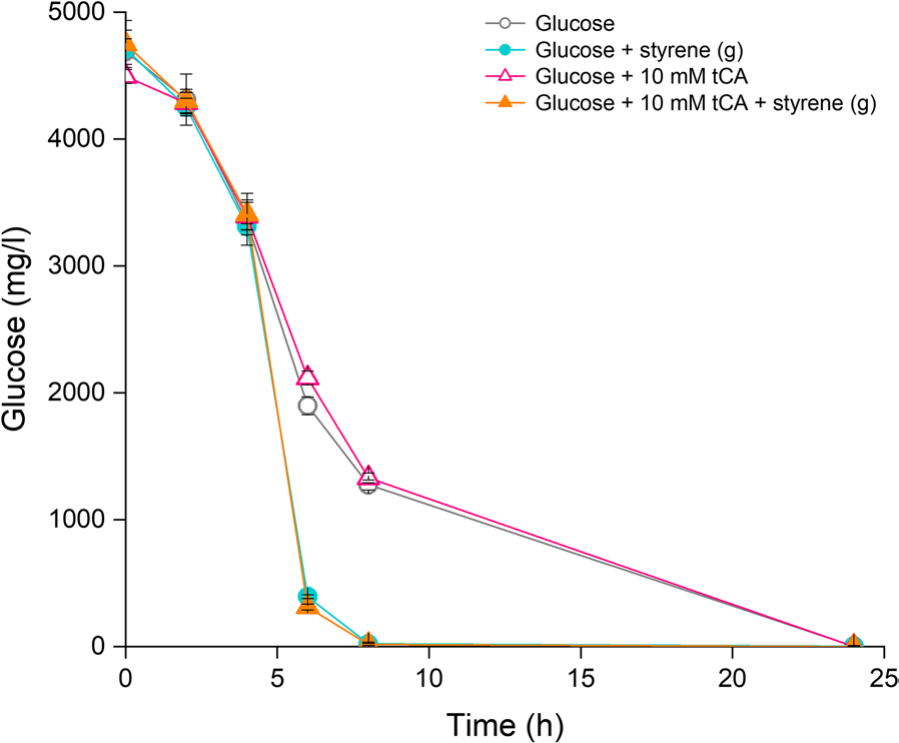
Glucose consumption by *P. putida* DOT-T1E in the absence and in the presence of non-metabolizable aromatic compounds. Control cells growing with glucose alone (grey open circles), glucose plus styrene in the gas phase (blue solid circles), glucose plus 10 mM *trans-*cinnamic acid (pink open triangles), or the two compounds simultaneously (orange solid triangles)

This common metabolic response to stressors may be related to the need to generate energy to overcome the negative effects of these molecules. In fact, we found that in cells growing with glucose as the sole carbon source, glucose was consumed at a higher rate in the presence of styrene regardless of the presence of *trans*-cinnamic acid (Fig. 3). Also, because the growth yield was lower when these aromatics were present, versus when glucose was the sole carbon source, we inferred that energy consumption is needed to cope with the toxicity.

Glucose is metabolized in *P. putida* through three convergent pathways that convert glucose into 6-phosphogluconate [58]. In response to the aromatic compounds, the porin OprB (involved in glucose uptake) and an ABC glucose transporter were induced by at least two-fold under all conditions (Additional file 6: Table S3). This agrees with the genetic organization of the *oprB* gene being part of the operon containing the ABC transporter [59]. We also found that the direct phosphorylation of glucose to glucose-6-phosphate catalyzed by glucose kinase (Glk) followed by glucose dehydrogenase (Zwf) was induced while the keto gluconate pathways was not. The final product of this branch for glucose metabolism is 6-phosphogluconate, which enters the Entner-Doudoroff pathway with EDD and EDA yielding glyceraldehyde-3-P and pyruvate. Our results show that the two steps of the Entner-Doudoroff pathway were induced. This is also consistent with the fact that *edd* belongs to an operon with the glucokinase gene (*glk)*; as well as with the fact that the *eda* gene is in an operon with *zwf* and *pgl* [59, 60] (Additional file 6: Table S3). Regarding repressed genes, we found that the genes for the biosynthesis of PQQ, the cofactor of gluconate dehydrogenase in the metabolism of glucose, was repressed as well as the gluconate transporter (Additional file 7: Table S4). These findings support the hypothesis that under aromatic compounds stress conditions the glucose-6-phosphate pathway becomes predominant. Therefore, under aromatic stress conditions it seems that glucose is preferentially assimilated through the glucose phosphorylative pathway.

Genes encoding Krebs cycle enzymes were induced between 2.0 and 4.9-fold regardless of the aromatic in the culture medium. Some of the genes were organized in clusters such as *suc* (T1E_0425 to T1E_0429), *sdh* and *icd/idh* genes (T1E_0537 and T1E_0538). Two citrate synthase genes (*glt*A and *prp*C) (T1E_0434 and T1E_5347) were also induced, leading to an increased ability to channel acetyl-CoA quickly and efficiently into the respiratory chain. Phosphoenolpyruvate carboxylase, which is involved in replenishment of the Krebs cycle intermediate OAA, was induced by about two-fold (Additional file 6: Table S3). In response to stress created by styrene (with and without *trans-*cinnamic acid), an NADH oxido-reductase was induced, together with a set of respiratory chain cytochromes (*nuoFIJKLM*) (T1E_4593, T1E_0717 to T1E_0720, T1E_0725 T1E_0824, T1E_0825, T1E_4136 to T1E_4145). The terminal CyoBCDE complex was also induced.

Along with the induction of an NADH-quinone oxidoreductase and a series of cytochromes, ATP synthase genes (*atpIBEFHAGDC)*, were also induced by 2.2- to 5.2-fold (Additional file 6: Table S3). Also related to energy use, genes in the urea cycle (*arc)* (T1E_1977 to T1E_1979) were induced 3- to sixfold (Additional file 6: Table S3). In accordance with these transcriptomic data, proteins levels that increased in response to aromatic compounds were those involved in glucose metabolism, the Krebs cycle and respiratory chains (NADH oxidases and cytochromes) (Additional file 6: Table S3).

Relevant to the induction Kreb’s cycle genes was our observed repression of the glyoxylate shunt, along with repression of certain genes involved in the respiratory chain (i.e., *yce, ped*), which would be expected to accommodate changes to the cellular carbon flux (Additional file 7: Table S4).

Other induced genes included a number of efflux pumps and stress response proteins, along with catabolic pathways for aromatics, which highly induced (see Additional file 6: Table S3). We found that in response to *trans*-cinnamic acid *ttgABC* genes (T1E_0243, T1E_0242 and T1E_0241) were induced between 2- and threefold, while *ttgDEF* (T1E_4281, T1E_4280, T1E_4279) were induced between 2 and fourfold in response to styrene*.* The greatest increase in protein levels corresponded to TtgGHI and TtgDEF efflux pumps, which were present at levels that were 2.5- and 3- fold higher than in the absence of aromatics (Additional file 8: Table S5).In addition to the above efflux pumps known to be involved in aromatic extrusion, we found two undescribed efflux pumps—*mtrABC* and *yhiUVW*—that were induced between 2.3- and 5.7-fold respectively (Additional file 6: Table S3). The specific substrates of these efflux pumps are unknown.

Dominguez-Cuevas et al. [60] reported that the stress response is needed to counteract the toxicity of aromatic compounds in *Pseudomonas*. Our observations confirm this by demonstrating that in the presence of only one aromatic compound (i.e., styrene or *trans-*cinamic acid), there was an induction of UspA family members (T1E_0387, T1E_2745, T1E_5564, T1E_5566, T1E_5582 and T1E_5584), alkylperoxidases (T1E_4718), catalases (T1E_3479), DUF domain proteins (T1E_1888), chaperones (i.e. ClpB (T1E_0798) and Hsp20 (T1E_5574) (Additional file 6: Table S3). When both aromatic compounds were present, they enhanced the stress imposed on the cells and in addition to all of the above mentioned defense systems, induced overexpression of a number of chaperones. This indicates that there is an additive effect when the two aromatic compounds are present. The chaperones that we found induced included the GroEL complex, SurA (T1E_2150), HtpG (T1E_2387) and GroSL (T1E_4380, T1E_4381), as well as enzymes related with general stress such as XenA/B (overexpressed by up to 7.7-fold) and, with oxygen stress such as glutathione transferases (T1E_0524, T1E_3573, T1E_4366) and a superoxide dismutase (T1E_1925), (Additional file 6: Table S3). The activation of ROS proteins may be related to the accelerated electron flux through the respiratory chain and the production of reactive oxygen species (ROS). For stress response genes, proteomic analyses agreed with transcriptomic data. We found heightened levels of alkylhydroperoxidases, glutathione oxidase (involved in quenching ROS), and protein folding chaperones (Additional file 6: Table S3 and Additional file 8: S5).

The differences that we observed between the three conditions included changes in the induction of regulators: 26 were altered in the presence of styrene, 8 with *trans-*cinnamic acid and 32 in the presence of both.

Although neither styrene nor *trans-*cinnamic acid can be metabolized by the cells, they induced certain catabolic pathways. It is known that a number of regulators exhibit an effector specificity than the catabolic pathway they regulate [61, 62]. In the presence of styrene the toluene dioxygenase pathway (*tod*) was induced, while in the presence of *trans*-cinnamic acid the *p*-cumate pathway was induced (Additional file 6: Table S3). In the presence of both compounds pathways for the degradation of toluene and *p*-cumate were induced, although the level of induction was lower with both aromatic compounds—a result that may indicate that effector competition is occurring, as has been described for the TodS/TodT system [62, 63].

Among the set of membrane proteins that were induced, a number of them are known to be involved in maintaining cell membrane structure. The conserved Tol-Pal trans envelope complex is important for outer membrane stability and cell division in Gram-negative bacteria [64]. It has been proposed that the Tol-Pal system mediates outer membrane constriction during cell division via cell wall tethering. For example, it has been shown that the TolB protein is involved in the maintenance of the *P. putida*’s cell membrane structure [65]. Related to this is the activation or overexpression of antiholins LrgA and LrgB, which were induced (3.7 to 5.0-fold) and which are thought to control peptidoglycan hydrolysis. Our findings implicate them in the stress response as modulators of membrane integrity.

We observed signs of active protein synthesis in the three conditions with stressors (Additional file 6: Table S3). This was likely an effort by the cell to replace damaged proteins, and the response was exacerbated when both aromatic compounds were simultaneously present (Additional file 6: Table S3). In response to styrene 18 genes involved in amino acid biosynthesis and 6 genes involved in protein synthesis were induced, suggesting a potential role for amino acid metabolism in styrene resistance. Also induced were *glnA* (glutamine synthetase) and *gltB* (glutamate synthase), which are needed for the incorporation of ammonium into organic carbon and its alternative channeling to amino acid biosynthesis. Regarding sulfur metabolism we found that the cysteine biosynthesis cluster (T1E_0565, T1E_0566 and T1E_4456 to T1E_4460) was induced when styrene and *trans-*cinnamic acid were present in the culture media (Additional file 6: Table S3).

We also found that catabolism of His and Phe was repressed in the presence of one or both of the aromatic compounds. Whether this results from the need of those amino acids for biosynthetic processes or repression due to their aromatic nature is unknown.

*Trans-*cinnamic acid, styrene and their combination resulted in the rather strong inhibition of a number of transporters. Some of these were found in operons related to the transport of amino acids (i.e., T1E_2666, T1E_2667, T1E_3208, T1E_0341, T1E_3315) and potassium (T1E_2369) (Additional file 7: Table S4). While some transporters were downregulated in the presence of *trans-*cinnamic acid (i.e., T1E_1254, T1E_3944, T1E_0320, T1E_0710, etc.), this was not the case for styrene (see Additional file 6: Table S4). The additive toxicity of *trans-*cinnamic plus styrene was apparent by the fact that a large number of transporters were repressed only when the two aromatic compounds were present (i.e., T1E_2543, T1E_4501, T1E_4503, T1E_4502). No other common patterns were found, but the list of all genes repressed in each condition that we tested are provided in Additional file 7: Table S4.

Proteomic analysis revealed a correlation between the upregulated genes and the proteins induced under the same conditions. In presence of styrene or styrene plus *trans*-cinnamic acid, there was increased levels of proteins related to amino acid metabolism, aromatic compound catabolic pathways, efflux pumps, Krebs cycle, cell membrane, transcriptional regulators, and stress response proteins (Additional file 8: Table S5).

### Mutational analyses to identify genes functionally relevant to stress responses induced by *trans*-cinnamic acid or styrene

In order to test the importance of genes implicated in the stress response through transcriptional data, mutational analysis were carried out. For a number of the genes that we identified, mutants in *P. putida* DOT-T1E were already available [66, 67]. We also constructed mutants related to Krebs cycle (SucC, PflU) and urea cycle (ArcA) (see Experimental Procedures).

**Fig. 4:**
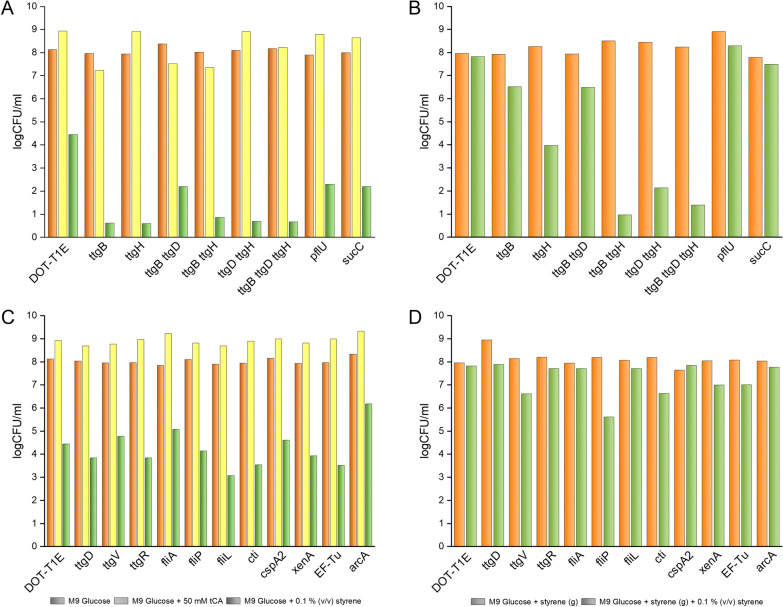
Survival of *P. putida* DOT-T1E mutants grown on glucose (**A** and **C**) or grown on glucose with styrene in the gas phase (**B** and **D**) after 60 min exposure to *trans-*cinnamic acid (50 mM) or styrene (0.1% (v/v)). Panel A and Panel B show mutants highly sensitive to a sudden styrene shock when grown on glucose, while C and D show mutants as sensitive as the wild-type strain to styrene when grown on glucose. Control without addition, orange bars; plus 50 mM *trans*-cinnamic acid, yellow bars; plus 0.1% (v/v) styrene, green bars

Using this collection, the survival of each of the mutants to shocks of 50 mM *trans-*cinnamic acid or 0.1% (v/v) styrene was tested. We found that when the cells were grown on glucose or glucose plus styrene in the gas phase, the sudden shock of 50 mM *trans-*cinnamic acid was well tolerated by all mutants tested (see Fig. 4A, C).

Styrene is significantly more toxic than *trans-*cinnamic acid, and this was deduced because a number of mutants were extremely sensitive to a sudden styrene shock, although toxicity varied based on growth conditions. In wild-type DOT-T1E grown on glucose, the sudden addition of 0.1% (v/v) styrene led to loss of viability by 4 orders of magnitude. In the absence of pre-adaption to hydrocarbons, mutants deficient in TtgB or TtgH, or both, the sudden addition of the chemicals resulted in a loss of viability by around 7 orders of magnitude. In this regard, it is worth mentioning that this mutant in terms of efflux pump is equivalent to KT2440 that exhibits the TtgABC efflux pump but not the other two efflux pumps identified here as relevant in tolerance to styrene, TtgDEF and TtgGHI. Under the same conditions, mutants deficient in PflU and SucC exhibited a 5 to 6 order of magnitude loss of cell viability (Fig. 4A). Mutants for TtgD, TtgV, TtgR, FliA, FliP, FliL, Cti, CspA2, XenA, ET-Tu and ArcA suffered a drop in viability in response to a sudden styrene shock similar to the wild-type (Fig. 4C). This suggests that the TtgABC and, in particular, TtgGHI are relevant in the response to styrene as well as the Krebs cycle enzymes.

When cells were pre-induced with low concentrations of styrene (i. e. in the gas phase), the most sensitive single mutant was TtgH, which experienced a viability drop by 4 orders of magnitude (Fig. 4B). Under the same conditions, the viability of TtgB mutant decreased by around 2 orders of magnitude. It should be noted that the double mutant *ttgB/ttgH* was extremely sensitive to styrene shock, while this extreme sensitivity was not observed for a *ttgB/ttgD* double mutant (Fig. 4B). No loss of viability was found with mutants in *sucC* or *pflU* when cells were pre-induced with styrene (Fig. 4B). Mutants for TtgD, TtgV, TtgR, FliA, FliP, FliL, Cti, CspA2, XenA, ET-Tu and ArcA pre-grown in the presence of styrene in the gas phase did not suffer a drop in viability in response to a sudden styrene shock similar to the wild-type (Fig. 4D). This suggests that TtgGHI is the most relevant pump to styrene extrusion, while TtgABC and TtgDEF play accessory roles. Given that the *ttgGHI* efflux pump genes are on a self-transmissible plasmid, we predict that the transfer of this pump to other chassis would be useful for the bioproduction of aromatic compounds.

## Discussion

Petrochemicals can be found all around us. They are components in the cars we drive, the fuels that power them, and are used to make packaging, textiles, paints, dyes, medical equipment, detergents and more. The manufacture of petrochemical compounds and their derivatives consume about 14% of the oil and gas used in the world [68] Petrochemicals provide us with an array of services, including sustenance, mobility and heating and cooling—services that encompass our universal needs as humans. Because we cannot replace goods produced from petrochemicals in the short-term, the contribution of the petrochemical industry to climate change is often overlooked and receives less attention than the transport and energy sectors [69].

Webb [70] indicated that our dependency on dwindling fossil resources and our growing awareness of the dangers posed by climate change are major driving forces for the transition towards the production of sustainable compounds from renewable feedstocks, such as lignocellulosic residues [71]. Lignocellulose can be processed to separate cellulose and hemicellulose polysaccharides from lignin. The former can be broken into their constituent sugars using enzymatic cocktails [71, 72]. Fermentation of the resulting monomeric sugars, which is known as a 2G process [9], can be carried out at room temperature using recombinant microbial chassis. This process can be used to produce value added chemicals and represents an approach that would have profound effects on the chemical industry by significantly reducing energy costs and CO_2_ emissions [5, 71].

We have concentrated our efforts on two aromatic compounds as potential bioproducts from 2G sugars; namely, styrene and *trans-*cinnamic acid. Styrene is one of the most used chemicals because it is employed in the production of polymers. The total global production of styrene in 2010 was 25 million tons. Currently, about 90% of the world’s production is based on the direct dehydrogenation of ethylbenzene under operating conditions that involve the use of temperatures above 600 ºC, iron oxide as a catalyst and a large amount of water vapor for heating purposes and reduction of coke formation [73]. Conventional styrene production processes consume 10 times as much energy as the production of similar chemicals (e.g., aromatic compounds such as benzene, toluene and xylene) and is, within the petrochemical sector, a major contributor to greenhouse gas emissions, including methane and CO_2_ [23].

Although it may not be widely acknowledged, styrene is a biogenic product. Styrene is naturally synthesized by plants and microbes from *L*-phenylalanine, and it has been found as a trace metabolite in cheeses, where it can cause an aroma defect that leads to consumer rejection. It has also been reported to be released by plants of the family *Styracaceae* [74]. McKenna and Nielsen [23] revealed that co-expression of a phenylalanine ammonia lyase and a *trans-*cinnamic decarboxylase in *E. coli* leads to styrene production, although production required the supply of *L*-phenylalanine exogenously because the production level was low, and maximum productivity was inhibited by styrene’s extreme toxicity. It has been recognised that the use of solvent-tolerant hosts may be a way to improve styrene biosynthesis [23, 24]. *Trans-*cinnamic acid derivatives such as cinnamaldehyde, cinnamyl alcohol, and hydro cinnamyl alcohol are widely used aroma compounds, with applications in the flavor, food and cosmetic industry, as well as use as antimicrobials, a nematicide and anti-inflammatory agents. Therefore, this chemical is another interesting target for biological production.

### *Pseudomonas putida*: a promising platform for aromatic chemical bioproduction

The effectiveness of various microbial hosts (e.g., *E. coli, Clostridium acetobutylicum, Corynebacteria* and yeasts) for the production of chemicals is often limited by the inherent sensitivity of the host to the chemical of interest. This is particularly true when synthetic pathways have been developed to produce aromatic hydrocarbons and medium chain alcohols such as butanol in *Clostridium* [75, 76]; isobutanol in *E. coli* and yeasts [77, 78] and aromatic compounds (e.g., 2-phenylethanol and styrene) in *E. coli* [11, 23].

Over the last 10 years, interest in using *P. putida* as a chassis for the production of industrially important chemicals (i.e., *p*-hydroxybenzoate or *p*-hydroxystyrene [79, 80] has significantly increased because of its advantages in robustness and metabolic versatility, and the fact that *Pseudomonas putida* is certified by NIH as a safe to manipulate microorganism [81]. Furthermore, a number of *P. putida* strains demonstrate resistance to aromatic compounds—in fact, some have been described to thrive in the presence of up to 90% toluene in double phase systems [31].

*Pseudomonas putida* DOT-T1E strain was isolated by our group in Granada and it represents, together with the S12 strain [54], the most organic solvent tolerant microorganism ever described. This makes it an ideal host for production of toxic chemicals such as aromatic hydrocarbons and aromatic amino acids. Previously, this strain was shown to be a suitable host for production of *p*-hydroxybenzoate from sugars in a joint project that we developed with Dupont [82]. Later, we showed that it can be used to produce phenylalanine from sugars [14].

In this study, we revealed the detailed physiological and biochemical responses of this strain to styrene and *trans*-cinnamic acid. Exposure to styrene or the aromatic acid activated a series of defensive tactics, some of which were ‘common’ in that they occurred after insult by a range of compounds (e.g., dyes, antibiotics and ROS generating compounds). These defensive tactics include the *cis–trans* isomerization of unsaturated fatty acids to strengthen the cell membrane [37, 52], and the synthesis of molecular chaperones that help to overcome the toxic effects of chemicals on unfolded proteins [31, 67]. We found that the presence of *trans-*cinnamic acid and/or styrene led to changes in bacterial metabolism, with a shift to the glucose phosphorylative pathway, an increase in Krebs cycle enzymes and the modification of the components of respiratory chains. As a consequence, higher respiration levels were observed, enabling extra energy to be generated in the presence of toxic compounds. The mechanism for extruding the toxic chemical(s) from the cell cytoplasm, periplasm and membrane were extremely important. Several RND family efflux pumps are key players in this process; specifically, TtgGHI (plasmid-encoded) and TtgABC (chromosomically encoded). In fact, mutants deficient in these efflux pumps were sensitive to the aromatic compounds and this sensitivity was exacerbated when both styrene and *trans-*cinnamic acid were simultaneously present.

These results are in contrast with a recent study by Machas et al. [83], which purports that the main response of *E. coli* at the transcriptional level involves: (i) general stress response (*rpoH* and *marA* gene); (ii) induction of membrane stability genes, such as those of phage shock protein response and (iii) cell membrane modification, including those required for peptidoglycan cross-linking layer with lipoproteins. However, the study reported no induction of efflux pumps transporters. Thus, while the two Gram-negative *E. coli* and *P. putida* respond to solvents by strengthening cell membranes, *P. putida* uses an additional weapon, the induction of efflux pumps to keep intracellular solvent concentrations.

Because *ttgGHI* genes are found on a self-transmissible plasmid that can be mobilized to other host platforms, such as the *E. coli* Gram-negative bacteria, our findings lay the foundation for new strategies to expand the range of toxic chemicals that other cell platforms can produce*.* Taken together, our findings provide deep insights that will help to advance the use of *P. putida* DOT-TIE and other microorganisms as aromatic chemical bioproduction chassis, and move us closer to achieving a more sustainable low carbon green chemistry future. From a biosynthesis point of view, our results suggest that a synthetic pathway that enables the efficient conversion of phenylalanine to styrene without the accumulation of *trans-*cinnamic acid would produce high styrene yields.

### Supplementary Information

**Additional file 1**: **Figure S1**. Tolerance of *P. putida* DOT-T1E to *trans-*cinnamic acid (0, 15, 25, 50, 75 and 90 mM) in A) M9 glucose plus 10 mM *trans*-cinnamic acid; B) M9 glucose plus styrene in the gas phase and C) M9 glucose, 10 mM *trans*-cinnamic acid and styrene in the gas phase. The results represent the average and standard deviation of three independent assays. Control, grey open circles or with the addition of *trans*-cinnamic acid and at 15 mM (blue solid circles); 25 mM (pink open triangles); 50 mM, (orange solid triangles); 75 mM, (green open squares); 90 mM, (purple solid squares).

**Additional file 2**: **Figure S2**. Survival of *P. putida* DOT-T1E, *P. putida *KT2440 and *E. coli* ET8000 grown on glucose (A) or grown on glucose with styrene in the gas phase (B) after 60 min exposure to styrene (0.1 % (v/v)). Control without addition, orange bars; plus 0.1 % (v/v) styrene, green bars.

**Additional file 3**: **Figure S3**. Volcano plot of *P. putida* DOT-T1E cells grown on M9 glucose plus 10 mM *trans*-cinnamic acid vs M9 glucose (A); M9 glucose with styrene in the gas phase vs M9 glucose (B); and M9 glucose plus 10 mM *trans-*cinnamic acid and styrene in the gas phase vs M9 glucose (C). Volcano plots showed that there were 973, 2200 and 2604 DEGs when cells were grown on styrene, *trans*-cinnamic acid or both compounds, respectively. The up-regulated genes were 548, 1137 and 1439, respectively, while 425, 1063 and 1165 were down-regulated genes.

**Additional file 4**: **Table S1**. Primers used in this study.

**Additional file 5**: **Table S2**. Number of genes and proteins uncharacterized or without known function up- or downregulated in *P. putida* DOT-T1E in response to *trans-*cinnamic acid (tCA), styrene supplied in the gas phase or both compounds.

**Additional file 6**: **Table S3.** Genes upregulated two-fold or more in response to *trans*-cinnamic acid (tCA), styrene supplied through the gas phase (Styrene (g)), or both compounds (tCA + Styrene (g)).

**Additional file 7**: **Table S4.** Genes downregulated two-fold or more in response to *trans*-cinnamic acid (tCA), styrene supplied through the gas phase (Styrene (g)), or both compounds (tCA + Styrene (g)).

**Additional file 8**: **Table S5.** Proteins induced 1.5-fold or more in response to styrene supplied through the gas phase (Styrene (g)), *trans*-cinnamic acid (tCA) or both compounds (Styrene (g) + tCA).

## Data Availability

All data are presented in the main body of the MS or as supplementary material. Biological material is available upon request.
