# Supplementary figures and images for: Insights into the susceptibility of Pseudomonas putida to industrially relevant aromatic hydrocarbons that it can synthesize from sugars
Source: Microb Cell Fact. 2023 Feb 2;22:22. doi: 10.1186/s12934-023-02028-y (PMC9893694; doi:10.1186/s12934-023-02028-y)

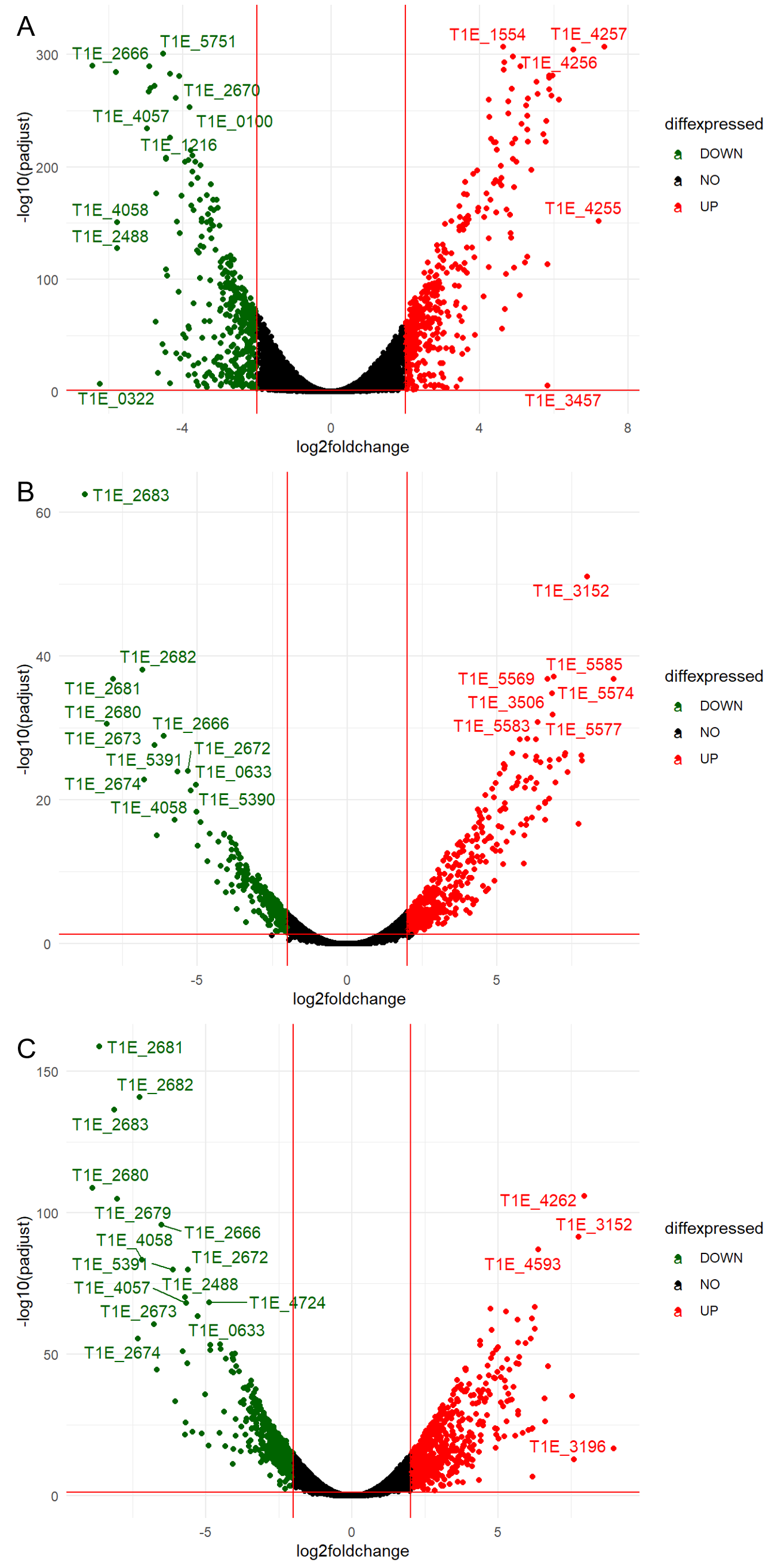

Supplement: Supplementary file 3 — Additional file 3: Figure S3. Volcano plot of P. putida DOT-T1E cells grown on M9 glucose plus 10 mM trans-cinnamic acid vs M9 glucose (A); M9 glucose with styrene in the gas phase vs M9 glucose (B); and M9 glucose plus 10 mM trans-cinnamic acid and styrene in the gas phase vs M9 glucose (C). Volcano plots showed that there were 973, 2200 and 2604 DEGs when cells were grown on styrene, trans-cinnamic acid or both compounds, respectively. The up-regulated genes were 548, 1137 and 1439, respectively, while 425, 1063 and 1165 were down-regulated genes. [file 12934_2023_2028_MOESM3_ESM.tif]
