# Supplementary material for: Insights into the susceptibility of Pseudomonas putida to industrially relevant aromatic hydrocarbons that it can synthesize from sugars
Source: Microb Cell Fact. 2023 Feb 2;22:22. doi: 10.1186/s12934-023-02028-y (PMC9893694; doi:10.1186/s12934-023-02028-y)
Supplement: Supplementary file 4 — Additional file 4: Table S1. Primers used in this study. [file 12934_2023_2028_MOESM4_ESM.docx]

| **Primer name** | **Sequence** |
| --- | --- |
| sucC3-F | CTGTAGTCGATCGTTCGAGCCG |
| sucC3-R | CAGGTTGACGATGTCCATGGT |
| pflU1-F | GTTCACGTAGGGGCCGCAGC |
| pflU1-R | CAGGTGCTCGGTTTCGGCAC |
| arcA1-F | GAACTGCTGTTCGACGATGTG |
| arcA1-R | GTGATGACCTCGATGCCGG |
