# Supplementary material for: Insights into the susceptibility of Pseudomonas putida to industrially relevant aromatic hydrocarbons that it can synthesize from sugars
Source: Microb Cell Fact. 2023 Feb 2;22:22. doi: 10.1186/s12934-023-02028-y (PMC9893694; doi:10.1186/s12934-023-02028-y)
Supplement: Supplementary file 5 — Additional file 5: Table S2. Number of genes and proteins uncharacterized or without known function up- or downregulated in P. putida DOT-T1E in response to trans-cinnamic acid (tCA), styrene supplied in the gas phase or both compounds. [file 12934_2023_2028_MOESM5_ESM.docx]

|  | **Genes** | | | **Proteins** | | |
| --- | --- | --- | --- | --- | --- | --- |
|  | **tCA** | **Styrene (g)** | **Styrene (g) + tCA** | **tCA** | **Styrene (g)** | **Styrene (g) + tCA** |
| **Up-regulated** |  |  |  |  |  |  |
| Uncharacterized | 0 | 29 | 13 | 40 | 72 | 96 |
| Unknown function | 159 | 99 | 138 | 1 | 7 | 3 |
| **Down-regulated** |  |  |  |  |  |  |
| Uncharacterized | 138 | 56 | 127 | 2 | 8 | 8 |
| Unknown function | 102 | 72 | 134 | 3 | 1 | 3 |

Filtrando proteómica por log > 1.5:

|  | **Genes** | | | **Proteins** | | |
| --- | --- | --- | --- | --- | --- | --- |
|  | **tCA** | **Styrene (g)** | **Styrene (g) + tCA** | **tCA** | **Styrene (g)** | **Styrene (g) + tCA** |
| **Up-regulated** |  |  |  |  |  |  |
| Uncharacterized | 40 | 72 | 96 | 0 | 5 | 3 |
| Unknown function | 157 | 98 | 138 | 0 | 0 | 0 |
| **Down-regulated** |  |  |  |  |  |  |
| Uncharacterized | 137 | 55 | 126 | 0 | 4 | 3 |
| Unknown function | 102 | 72 | 134 | 0 | 1 | 1 |
